# Investigation of the chemical properties of *Mentha pulegium* essential oil and its application in *Ocimum basilicum* seed mucilage edible coating for extending the quality and shelf life of veal stored in refrigerator (4°C)

**DOI:** 10.1002/fsn3.2522

**Published:** 2021-08-17

**Authors:** Hadi Tanavar, Hassan Barzegar, Behrooz Alizadeh Behbahani, Mohammad Amin Mehrnia

**Affiliations:** ^1^ Department of Food Science and Technology Faculty of Animal Science and Food Technology Agricultural Sciences and Natural Resources University of Khuzestan Mollasani Iran

**Keywords:** edible coating, essential oil, mucilage, natural preservative, shelf life

## Abstract

Nowadays, the tendency toward the application of natural preservatives to extent the shelf life of food products has grown. The purpose of the present research was to evaluate the effect of the basil seed mucilage (BSM)‐based edible coating containing different concentrations of *Mentha pulegium* essential oil (MPEO) on the shelf life of the veal stored at refrigerator temperature. Firstly, the chemical composition and functional groups of MPEO were detected through gas chromatography–mass spectrometry (GC‐MS) and Fourier transform infrared spectroscopy (FTIR). Then, the BSM‐based edible coatings containing 0%, 0.5%, 1%, 1.5%, and 2% MPEO were prepared, and the veal samples were coated with them. The physicochemical, microbial, and sensory properties of the samples were investigated during the 9‐day storage period at 4°C. Twenty‐five compounds were detected in MPEO with limonene being the major one (28.44%). The results revealed that the lightness, hardness, and moisture content of the samples decreased during storage. The coating containing the essential oil could properly restrain the rise in pH, peroxide value (PV), and thiobarbituric acid value (TBA). Based on microbial analyses, the shelf life of the coated sample without the essential oil and those containing 0.5%, 1%, 1.5%, and 2% of the essential oil were, respectively, extended up to 3, 6, 9, 9, and 9 days relative to the control. Moreover, the coating containing the essential oil produced no unfavorable effect on the sensory properties of the meat samples. In conclusion, the BSM‐based edible coating containing different concentrations of MPEO can be applied as a natural preservative to enhance the resistance of meat products against microbial spoilage and fat oxidation.

## Graphical abstract

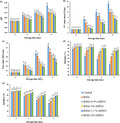

## 1 INTRODUCTION

Owing to its abundant nutrients, red meat is one the major food products consumed by humans. Given the high perishability of meat, the hygiene and shelf life of this product are of great importance to producers and consumers. Up to now, various methods, including smoking, drying, and storage at low temperatures have been utilized to extend the shelf life of meat and its products. Application of edible films and coatings is one of the novel technics for the storage of meat and its products (Dave & Ghaly, 2011).

Anti‐microbial edible films and coatings containing natural essential oils and extracts are considered to be a novelty in food packaging (Shen et al., 2010). Characteristics, such as biodegradability, gradual release of the active compounds present in the coating, minimizing the moisture loss of food products, and preventing the release of aromatic compounds are among the benefits of employing this technic of storage (Wang et al., 2010). Utilization of herbal essential oils and extracts with anti‐microbial and anti‐oxidative effects in edible coatings can be a suitable solution for inhibiting the microbial and chemical spoilage of food products (Alizadeh Behbahani et al., 2017c).

*Mentha pulegium* L. belongs to the family of Lamiaceae and the genus of Mentha (Khezri et al., 2020). It can be found in the wet regions of Europe, North Africa, Australia, North America, and Asia (Soilhia et al., 2019). The height of some shrubs of this perennial herbaceous plant reaches one meter. Due to its desirable aroma and properties, *Mentha pulegium* is used as a seasoning and preservative in the food industry (Ahmed et al., 2018). The aerial segments of this medicinal plant are employed in traditional medicine for the treatment of bronchitis, sinusitis, tuberculosis, indigestion, diarrhea, flatulence, and vomiting (Gülçin et al., 2020). Menthone, pulegone, and isomenthone are the major components of *Mentha pulegium* (Khezri et al., 2020).

Basil (*Ocimum basilicum* L.) belongs to the Lamiaceae family. Nowadays, it is cultivated in several regions of the world. At the same time, India and Asia are known as its primary origin (Ahmed et al., 2019). This plant absorbs water through the outer segments of its seeds. Glucomannan (43%), xylan (24%), and uric acid (6%) are the major constituents of BSM (Tantiwatcharothai & Prachayawarakorn, 2019). In the structure of the mucilage, glucomannan and xylan act as the hydrophobic and hydrophilic moieties, respectively. Xylan causes swelling by water absorption (Nourozia & Sayyari, 2019). Biodegradability, low‐cost production, easy extraction, high water absorption, temperature resistance, and favorable rheological properties have caused BSM to find its application in the preparation of edible films and coatings (Keisandokht et al., 2018; Majdinasab et al., 2020).

The objective of this study was to produce edible coatings based on BSM and MPEO, as well as investigating the physicochemical, microbial, and sensory properties of the coated veal samples during refrigerated storage for 0, 3, 6, and 9 days.

## 2 MATERIALS AND METHODS

### 2.1 Chemicals and reagents

The culture media, including plate count agar, mannitol salt agar, eosin methylene blue, and yeast extract glucose chloramphenicol agar were supplied from Conda pronadisa. Chloroform, acetic acid, potassium iodide, potassium sulfate, TBA indicator, and trichloroacetic acid were purchased from Sigma‐Aldrich.

### 2.2 MPEO preparation

The essential oil was bought from Johareh Tam Mashhad, which had been extracted through water distillation.

### 2.3 Preparation of basil seed mucilage

BSM was extracted according to the method previously optimized by Razavi et al. (2009). To that end, basil seeds were firstly macerated in deionized water at a ratio of 1:50, 50°C and pH = 7. After 10 min, the mixture was centrifuged at 5000 *g*, and the mucilage was extracted which was further bleached with ethanol 96% at a ratio of 1:3. It was then freeze‐dried (OPERON. FDB‐5503) and stored in a package impervious to moisture.

### 2.4 Detection of the chemical compounds of MPEO

GC‐MS was employed to detect the constituents of the essential oil. One microliter of the essential oil was injected into a gas chromatograph (Agilent Technologies 7890 A) equipped with a column, 30 m in length, 0.25 mm in internal diameter, and 0.25 µm in film thickness (Agilent Technologies Inc., HP‐5 MS) and integrated with a mass spectrometer (Agilent Technologies 5975). The initial temperature of the column was set at 40°C which was elevated up to 250°C at 2.5°C/min. Helium was used as the carrier gas at a flow rate of 1.1 ml/min, and the ionization energy was 70 eV. The chemical compounds of MPEO were detected using the spectra and retention indices of n‐alkanes along with referring to the database of natural compounds (Alizadeh Behbahani et al., 2020).

### 2.5 Examination of MPEO functional groups

Fourier transform infrared spectroscopy (FTIR) was employed to identify the functional groups of the MPEO. First, the essential oil powder was mixed with potassium bromide and pressed to be converted into a tablet. The essential oil spectrum was recorded in the wavenumber range of 4000–400 cm^−1^ using a spectrometer (Avatar 370, Thermo Nicolet; Alizadeh Behbahani, Falah, et al., 2020; Falah et al., 2021).

### 2.6 Veal chemical composition

The moisture, ash, fat, and protein contents of the veal samples were quantified according to AOAC (2005).

### 2.7 Edible coating preparation and coating of veal samples

In order to coat the veal samples, 1.5 g of the BSM and 0.52 g Tween 80 (35% of the dry weight of the BSM) were made to volume and blended with 100 ml of sterile distilled water. The solution temperature was set at 45–50°C. MPEO, as an ingredient with antibacterial effect, was added to the BSM solution at 0%, 0.5%, 1%, 1.5%, and 2%. Fresh veal samples were macerated and coated for 1 min in the solution containing the BSM and various concentrations of the essential oil. Four samples coated with the BSM and different concentrations (0.5%, 1%, 1.5%, and 2%) of the essential oil, one sample free of both the essential oil and the BSM, and one sample coated with the BSM without the essential oil were stored at refrigerator temperature for 9 days. The physicochemical, microbial, and sensory properties of the veal samples were investigated at certain time intervals (Heydari et al., 2020).

### 2.8 Veal chemical analyses

#### 2.8.1 pH

In order to determine the pH of the samples, 5 g of each sample was mixed with 45 ml of distilled water and homogenized. The pH values of the samples were measured using a digital pH meter at room temperature (Xiong et al., 2020).

#### 2.8.2 Peroxide value

In order to extract the lipid content of the samples, 60 ml of methanol was added to 15 g of the veal sample in a separatory funnel. After homogenization, 30 ml of chloroform was also added. After 5 min, another 30 ml of chloroform was added, and the solution was set aside at ambient temperature for 24 h so the sample lipid content would be extracted. Finally, 36 ml of distilled water was added to the contents of the separatory funnel, and the lower phase was separated after 2 h. The solvent was evaporated using a rotary evaporator, and only the sample lipid remained. For the determination of peroxide value (PV), a certain amount of the extracted lipid, 25 ml of acetic acid–chloroform (chloroform: acetic acid ratio of 2:3), 0.5 ml of saturated potassium iodide, 30 ml of distilled water, and 0.5 ml of starch 1% were mixed in a 250‐ml Erlenmeyer flask. The released iodine was titrated with sodium thiosulfate 0.01 N. PV was expressed as meq O_2_/kg (Majdinasab et al., 2020).

#### 2.8.3 Thiobarbituric acid value

First, 2 g of the veal samples was homogenized with 5 ml of trichloroacetic acid 20% for 2 min. The resulting mixture was filtered using a filter paper. Five milliliter of the filtrate was blended with 5 ml of TBA indicator 10 mM in a test tube. The tube was kept in a water bath at 100°C until color change was observed (about 1 h). After cooling down the tube, the absorbance value of the solution was measured at 532 nm (Lashkari et al., 2020).

### 2.9 Physical analyses of veal

#### 2.9.1 Moisture content

For quantifying the moisture content of the samples, they were heated in a hot‐air oven at 105°C until reaching a constant weight (5 h) (Barzegar et al., 2020).

#### 2.9.2 Texture analysis

A texture analyzer (TA, XT2i, the UK) was used to examine the hardness of the meat samples. During the test, the sample cubes (2*2*2 cm^3^) were compressed up to 30% of their initial height by the aluminum probe with a diameter of 36 mm (36 p/36). The probe speed was set at 5 mm/s (Heydari et al., 2020).

#### 2.9.3 Colorimetry

The color indices of the veal samples, namely L* (lightness), a* (redness), and b* (yellowness) were measured using a colorimeter (Konica Minolta, CR‐400, JP). The total color difference (Δ*E*) between the samples was calculated using the Equation (1) (Noshad et al., 2021):

(1) $$\Delta E=\sqrt{{\left(L^{*}\_L\right)}^{2}+{\left(a^{*}\_a\right)}^{2}+{\left(b^{*}\_b\right)}^{2}}.$$

### 2.10 Microbiological analysis

The microbial load of the veal samples stored at 4°C was assessed at certain time intervals (days 0, 3, 6, and 9). The stock solution was prepared by mixing 5 g of the veal and 45 ml of sterile physiological serum and homogenized for 1 min. Subsequent sequential dilutions were prepared using the stock solution. In all the steps before dilution, the solutions were homogenized for 1 min. Eventually, 100 ml of each solution was poured onto the culture media and spread with an L‐shaped rod. The performed microbial tests were as follows:

Total viable count was done using plate count agar and incubation at 37°C for 24 h.
Psychrotrophic count was carried out using plate count agar and incubation at 20°C for 24 h.
Mold, yeast, and fungi count were performed using yeast extract glucose chloramphenicol agar and incubation at 27°C for 72 h.
*Staphylococcus aureus* count was conducted using mannitol salt agar and incubation at 37°C for 24 h.
*Escherichia coli* count was done using eosin methylene blue and incubation at 37°C for 24 h (Kiarsi et al., 2020).

### 2.11 Sensory evaluation

The color, odor, texture, and overall acceptability of the samples were assessed on the test days. The veal samples were placed on the plates coded with random three‐digit numbers. Nine‐point hedonic scale (1 = the lowest quality and 9 = the highest quality) was applied to determine the quality of the samples. The samples with scores less than 4 were considered unacceptable (Barzegar et al., 2020).

### 2.12 Statistical analysis

The obtained data were analyzed through one‐way analysis of variance (ANOVA) using SPSS version 16. Duncan's multiple range test was employed for mean comparison at 95% confidence level. All the experiments were triplicated.

## 3 RESULTS AND DISCUSSION

### 3.1 Chemical composition of MPEO

Twenty‐five substances were detected in the MPEO using GC‐MS (Table 1). Limonene (28.44%), D‐carvone (18.76%), eucalyptol (8.86%), and pulegone (8.65%) constituted the major compounds of the essential oil.

**TABLE 1:** Chemical composition of *Mentha pulegium* essential oil

No	Compounds	Retention time (min)	%	Chemical formula
1	α‐Pinene	6.94	3.26	C_10_H_16_
2	Camphene	7.33	0.44	C_10_H_16_
3	Sabinene	8.04	2.07	C_10_H_16_
4	β‐Pinene	8.13	4	C_10_H_16_
5	β‐Myrcene	8.55	1.46	C_10_H_16_
6	Limonene	9.74	28.44	C_10_H_16_
7	Eucalyptol	9.80	8.86	C_10_H_18_O
8	γ‐Terpinene	10.54	0.21	C_10_H_16_
9	Linalool	11.81	0.63	C_10_H_18_O
10	Menthone	13.36	1.75	C_10_H_18_O
11	Borneol	13.73	0.55	C_10_H_18_O
12	Levomenthol	13.95	6.02	C_10_‐H_20_O
13	Neodihydrocarveol	14.60	6.45	C_10_H_18_O
14	Pulegone	15.86	8.65	C_10_H_16_O
15	D‐Carvone	16.06	18.76	C_10_H_14_O
16	Bornyl acetate	17.08	0.17	C_12_H_20_O_2_
17	Dihydrocarvyl acetate	18.22	0.49	C_12_H_20_O_2_
18	β‐Bourbonene	19.71	0.94	C_15_H_24_
19	Caryophyllene	20.61	3.09	C_15_H_24_
20	α‐Humulene	21.46	0.41	C_15_H_24_
21	Epi‐bicyclosesquiphellandrene	21.69	0.26	C_15_H_24_
22	Germacrene	21.78	0.45	C_15_H_24_
23	Bicyclogermacrene	22.52	0.13	C_15_H_24_
24	Naphthalene	22.94	0.2	C_10_H_8_
25	Caryophyllene oxide	24.62	0.21	C_15_H_24_O

Ahmed et al. (2018) examined the chemical composition of the MPEO native to Egypt. In their research, pulegone (57.8%), menthone (9.5%), and limonene (6.9%) were the major detected compounds. Saadat (2018) introduced pulegone, cineol, and menthofuran as the main components of MPEO. Many researchers have attributed these variations to climate conditions, time and type of cultivation, soil chemical composition, plant species, harvest season, and the methods of drying and essential oil extraction.

### 3.2 MPEO functional groups

**FIGURE 1:**
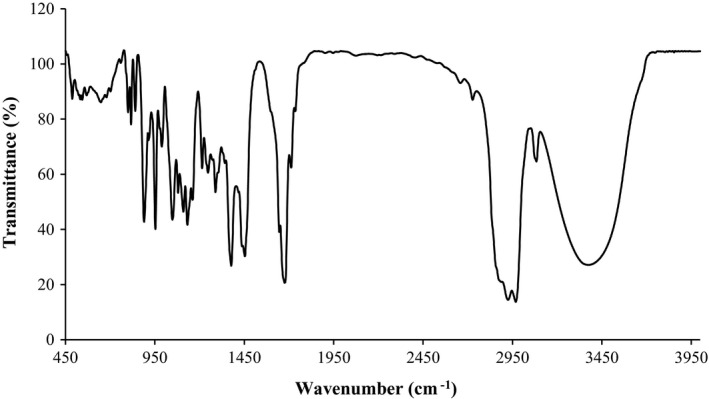
FTIR spectrum of *Mentha pulegium* essential oil

The FTIR spectrum of the MPEO is depicted in Figure 1. Based on the obtained spectrum, the principal peaks of the essential oil were observed at 3376.15, 3084.18, 2968.83, 2925.47, 1675.14, 1453.41, 1377.11, 1288.67, 1247.14, 1214.37, 1131.87, 1109.69, 1079.93, 1048.03, 988.49, 952.85, 889.40, 840.46, 816.95, 800.18, and 648.54 cm^−1^.

The FTIR results demonstrated that the band in the wavenumber range of 3400–3000 cm^−1^ was associated with the stretching vibrations of the O–H groups of water molecules, as well as those of the phenolic and alcoholic compounds of the essential oil like menthol (Alavi et al., 2021). The peak at 2968.83 cm^−1^ was ascribed to the asymmetric stretch of C–H and CH_3_ groups (Petrakis et al., 2009). In addition, the peaks at 2925.47 cm^−1^ belonged to the asymmetric stretch of CH_2_ and the symmetric stretch of CH_3_. The peak appeared at 1675.14 cm^−1^ was related to the carbonyl (C=O) groups of piperitone and pulegone, 1453.41 cm^−1^ to CH_2_ scissoring and the asymmetric deformation vibrations of CO–CH_2_, 1377.11 cm^−1^ to CH–CH_3_ symmetric deformation vibrations present in isomenthone and pulegone, and 1247.14 cm^−1^ to alkyl C–N stretch (Kanakis et al., 2012; Petrakis et al., 2009). The peak emerged at 1214.37 cm^−1^ may be due to the symmetric deformation of C–H (Kanakis et al., 2012). It should be noted that the wavenumbers of 988.49 and 952.85 cm^−1^ can be attributed to the bending C–H of alkenes (Al‐Shareefi et al., 2019). Overall, the peaks occurred in the range of 1131–936 cm^−1^ belonged to the vibrations of the CH_3_ groups of pulegone. Moreover, the range of 1500–500 cm^−1^ pertains to the finger print area and is unique (Petrakis et al., 2009).

Al‐Shareefi et al. (2019) evaluated the functional groups of *Mentha pulegium* methanolic extract through FTIR. The results indicated the presence of alkenes, alkyl halides, and aromatic compounds. In the research conducted by Bovandi et al. (2020) on *Mentha pulegium* extract, a broad band peaked at 3402 cm^−1^ was observed which was believed to be associated with the O–H groups of the phenolic compounds. In our study, the band ranging from 3400 to 3000 cm^−1^ was related to the O–H groups of the phenolic and alcoholic compounds of the essential oil.

### 3.3 Veal chemical composition

The chemical composition of the veal used in the present research is summarized in Table 2. AOAC standard methods were utilized to determine the meat chemical composition.

**TABLE 2:** Chemical composition of veal

Composition	%
Moisture	65.85 ± 0.43
Protein	18.3 ± 0.3
Ash	1.92 ± 0.15
Fat	14.2 ± 0.26

### 3.4 Chemical analysis

#### 3.4.1 pH

**FIGURE 2:**
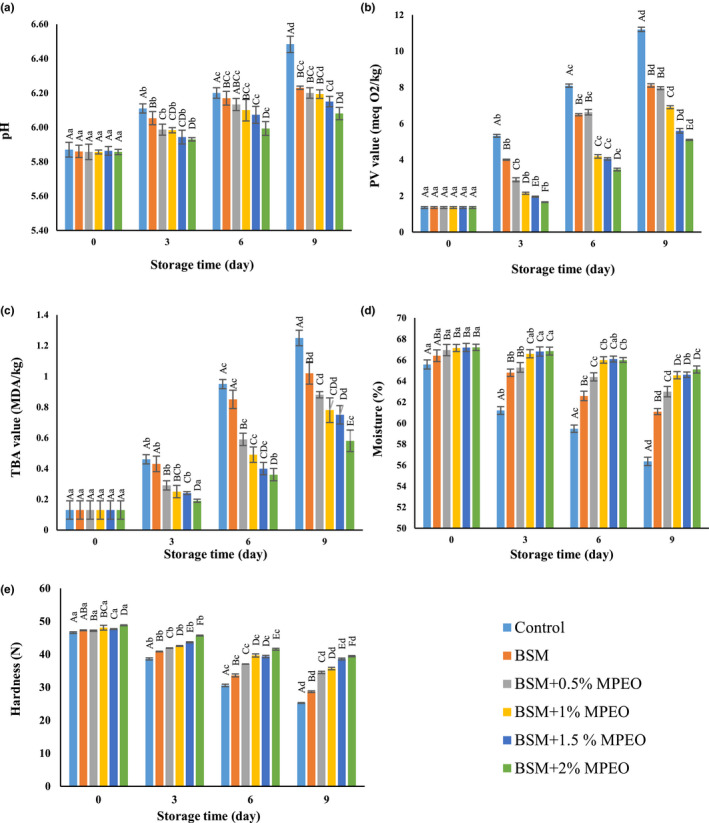
Changes in pH (a), PV (b), TBA (c), moisture content (d), and hardness (e) of the veal samples during refrigerated storage

The effect of pH change on the veal samples is depicted in Figure 2a. ANOVA showed that the pH of all the samples significantly increased during storage; however, the elevation slope was gentler in the coated samples relative to the control. The effect of coating was observed even on the first day of storage such that the pH of the control was more than that of the other samples, but this difference was not significant at 95% confidence level. The highest and lowest pH values on the 9th day of storage belonged to the coated sample containing 2% of the essential oil and the control, respectively, which were significantly different at *p* < .05.

Cattle age, sex, and diet in addition to the pre‐slaughter stresses are among the most important factors influencing the meat initial pH. It has been cited in the literature that bacteria play a key role in pH increase in such a way that bacterial enzymes give rise to pH by dissociating proteins and producing nitrogenous substances, such as ammonia (Lashkari et al., 2020). On the other hand, microbial activities cause an increase in carbon dioxide. The coating acts as a barrier to the expulsion of carbon dioxide. As the gas concentration increases, it converts into carbonic acid and subsequently, pH decreases. The gentler slope of pH increase in the coated samples compared with the control could be ascribed to this fact. The rise in the essential oil percentage also restrained intense pH variations, which is attributed to the antimicrobial properties of the essential oil (Ruan et al., 2019). Furthermore, the essential oil affected the solubility of carbon dioxide and reduced the coating permeability to this gas. This led to the accumulation of carbon dioxide inside the coating, pH reduction, and a decrease in the growth of microorganisms (Alizadeh Behbahani et al., 2017).

Alexandre et al. (2020) applied edible coatings containing herbal essential oils and extracts to extend the shelf life of meat products. These researchers cited microbial activities, CO_2_ concentration increase, and the degradation of nitrogenous compounds as the main reasons behind the rise in pH, conforming to the findings of the present study.

#### 3.4.2 Peroxide value

The changes in the PV of the veal samples are depicted in Figure 2b. The PV of all the samples was equal to 1.36 meq O_2_/kg on the first day. The slope of the PV increase was steeper for the control than for the other samples. In contrast, the PV of the coated sample containing 2% of the essential oil showed the least variations and increased from 1.36 meq O_2_/kg on the first day up to 5.1 meq O_2_/kg on the 9th day. Utilization of the coating and the rise in the essential oil concentration inhibited dramatic changes in the PV so that the coated sample containing 2% of the essential oil had the lowest PV on the 3rd, 6th, and 9th days.

7 meq O_2_/kg is the permitted PV for meat samples (Alizadeh Behbahani & Imani Fooladi, 2018a), whereby the PV of the control was more than this limit since the 6th day. Moreover, the PVs of the coated sample without the essential oil and the coated one containing 0.5 of the essential oil exceeded this permitted limit since the 9th day of storage.

Owing to their high content of unsaturated fatty acids, meat products are highly prone to chemical spoilage. Hydroperoxides are considered the major products of the initial stage of oxidation. They are odorless and are not usually detected by consumers (Lashkari et al., 2020). Like a barrier, the BSM‐based coating seems to have lowered the contact between oxygen and the sample surfaces and disrupted oxidation. Additionally, there was an inverse correlation between PV and the essential oil concentration. Previous studies have pointed to the phenolic and flavonoid contents, and the proper antioxidant activity of MPEO (Fatiha et al., 2015; Politeoa et al., 2018). Consequently, the delay in the oxidation of the samples with high concentrations of the essential oil could be due to the phenolic and flavonoid compounds as well as the antioxidant activity of MPEO (Rezaeifar et al., 2020).

Majdinasab et al. (2020) accomplished similar results in examining the application of BSM in extending the shelf life of chicken fillet.

#### 3.4.3 Thiobarbituric acid value

The changes in the TBA value of the samples during storage are illustrated in Figure 2c. The TBA value of the samples was equal to 0.13 MDA/kg meat on the first day. The results indicated that there was direct correlation between the TBA value and the storage time. The increase rate of the TBA value of the control was more than that of the coated samples and reached 1.29 MDA/kg meat on the 9th day of storage. ANOVA demonstrated that the TBA value of the control significantly (*p* < .05) differed from that of the coated samples on the 6th and 9th days.

The acceptable and permitted TBA value is 1 MDA/kg for meat products (Alizadeh Behbahani & Imani Fooladi, 2018a). Therefore, the TBA values of the control and the coated sample free of the essential oil exceeded the permitted limit on the 9th day of storage.

The hydroperoxides produced in the first stage of oxidation are unstable and degraded under the influence of various factors. Aldehydes are known as the main products of the second stage of oxidation, which reacts with the TBA indicator and develop a color complex (Majdinasab et al., 2020). Similar to PV, the coating acted as a barrier to oxygen penetration in this stage and restricted the oxidation of the coated samples relative to the control. Furthermore, the results revealed that the essential oil concentration was indirectly correlated with the TBA value, which could be caused by the phenolic compounds and antioxidant activity of MPEO (Alexandre et al., 2020; Shin et al., 2017).

Ragab Abdallah et al., (2018) reported similar results for the application of edible coatings in extending the beef shelf life. They also mentioned the role of the coating in preventing the oxygen penetration.

### 3.5 Physical analysis

#### 3.5.1 Moisture content

The variations in the moisture content of the veal samples are shown in Figure 2d. The moisture contents of all the samples lowered during storage so that they were significantly (*p* < .05) different from one another on various days. The control experienced the most changes, and its moisture content decreased from 65.58% (first day) to 56.36% (9th day). In contrast, the coated sample containing 2% of the essential oil showed the least variations, and its moisture content was reduced from 67.21% (first day) to 65.1% (9th day).

In general, it can be declared that coating inhibits the moisture loss of meat samples. The effect of coating was considerable even on the first day of storage, and the moisture content of the control was significantly (*p* < .05) different from that of all the coated samples. Some researchers believe that the coating acts as a physical barrier because of its low permeability to water vapor and brings about the retention of the meat sample moisture content (Feng et al., 2019; Kiarsi et al., 2020). On the other hand, as the essential oil concentration was raised, moisture loss decreased. Nisar et al., (2019) pertained this to the hydrophobicity of essential oils, which cause a reduction in the number of hydrophilic groups. Noshad et al. (2021) acquired similar findings for the moisture content of the coated buffalo meat.

#### 3.5.2 Texture analysis

The hardness values of the veal samples during storage are demonstrated in Figure 2e. Based on the obtained data, the hardness of the samples and the storage time were inversely correlated. The increase in the essential oil concentration as well as coating elevated the hardness of the samples, compared with the control. The largest hardness reduction was associated with the control, which decreased from 46.59 N on the first day to 25.33 N on the 9th day. The highest hardness (48.8 N) belonged to the coated sample containing 2% of the essential oil on the first day of storage.

Texture is one of the most important physical properties of meat and is regarded as a quality indicator among consumers. Genus, species, and stress are among the pre‐slaughter effective factors, and the amount of the connective tissue, water‐holding capacity, and pH are the most influential post‐slaughter factors on the meat quality (Juarez et al., 2012). It has been mentioned in the literature that the breakdown of collagen and myofibrillar proteins by microbial enzymes such as collagenases, calpains, and cathepsins brings about the destruction of the meat texture and a decrease in its hardness. At the same time, the MPEO, with an appropriate antimicrobial effect, restrained microbial activities, thus preventing the massive reduction in the hardness of the coated samples containing the essential oil, as compared with the control (Xiong et al., 2020). In addition, water‐holding capacity has direct and inverse correlations with pH and hardness, respectively. As a result, it can be claimed that during storage, the pH and water‐holding capacity of the samples rose, while their hardness was reduced (Heydari et al., 2020). The findings of the present study are in agreement with those achieved by Barzegar et al. (2020) and Xiong et al. (2020).

#### 3.5.3 Colorimetry

**FIGURE 3:**
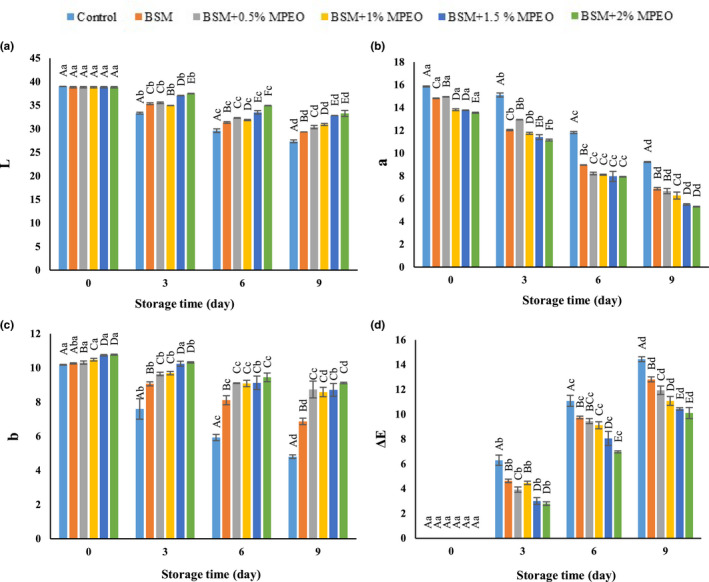
Changes in L* (a), a* (b), b* (c), and Δ*E* (d) of veal samples during 9 days of storage at 4°C

The changes in the L* of the veal samples during storage are demonstrated in Figure 3a. This color index represents the lightness of the samples. According to the obtained data, the lightness of the control was more than that of the coated samples on the first day; however, this difference was not significant (*p* > .05). As time went by, the lightness of all the samples significantly lowered which was more pronounced for the control than for the other samples. Meat color is considered a primary attribute attracting consumers’ attention and is an indicator of the meat freshness from the consumers’ points of view (Chaparro‐Hernández et al., 2019). The lower lightness of the coated samples, relative to the control, on the first day is likely related to the darkness of the coating. Many researchers believe that the decrease in the lightness of the samples during storage is owing to oxidation, alteration of protein structures, and increased light scattering (Alizadeh Behbahani et al., 2020; Lashkari et al., 2020). Moreover, the less pronounced reduction in the lightness of the coated samples, compared with the control, is associated with the antioxidant properties of the MPEO in addition to the inhibition of oxygen penetration by the coating, which controlled oxidation (Vital et al., 2016).

The alterations in the a* of the veal samples during storage are presented in Figure 3b. This color index denotes the redness of the samples. It was significantly reduced in all the samples during storage. The reduction slope was gentler for the control rather than the coated samples. Meat redness is affected by several factors, including myoglobin content, texture pH, and the presence of antioxidants (Mancini & Hunt, 2005). The decreased redness may be due to the conversion of oxymyoglobin (bright pink) to metmyoglobin (brown). The presence of the coating caused a reduction in oxygen pressure, thus inhibiting the production of oxymyoglobin (Kiarsi et al., 2020; Ruan et al., 2019).

The variations in the b* of the meat samples during storage are summarized in Figure 3c. This color index stands for yellowness. The b* of all the samples significantly lowered during storage. The changes in the b* of the control were more pronounced compared with the coated samples. In the literature, the smaller reduction in the yellowness of coated samples and the coated ones containing an essential oil has been attributed to the color of the coating and the essential oil (Kiarsi et al., 2020; Lashkari et al., 2020).

**FIGURE 4:**
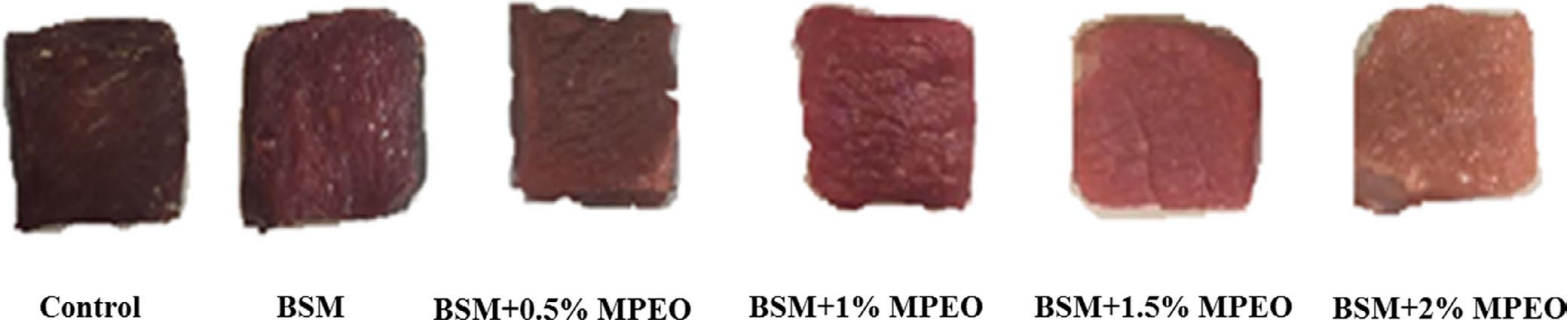
Effect of different concentrations of *Mentha pulegium* essential oil added to Basil seed mucilage (BSM)‐based edible coating on color changes of veal samples during 9 days of storage at 4°C

The total color change of the samples was evaluated using Δ*E* which was computed using L*a*b* (Figure 3d). The most and least changes, respectively, belonged to the control and the coated sample containing 2% of the essential oil. The results of this research are consistent with those reported by Khare et al., (2016) and Guerrero et al., (2020) (Figure 4).

### 3.6 Microbiological analysis

#### 3.6.1 Total viable count

**FIGURE 5:**
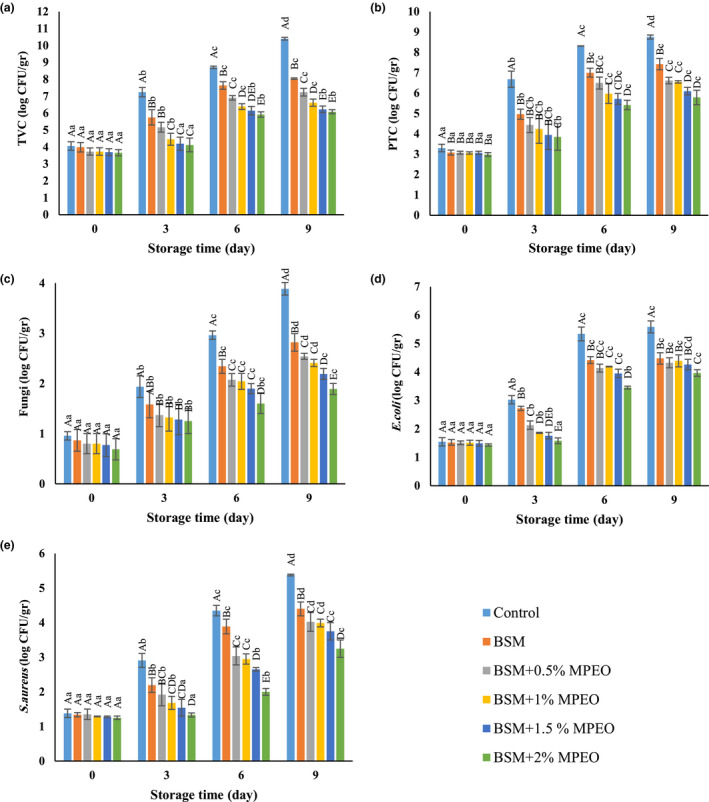
Changes in total viable count (a), psychrotrophic count (b), fungi count (c), *Escherichia coli* count (d), and *Staphylococcus aureus* count (e) of the veal samples stored at 4°C for 9 days

The results of the total viable count of the veal samples during refrigerated storage are shown in Figure 5a. The initial total viable count ranged from 3.66 to 4.06 log CFU/g. There was no significant difference between the samples at *p* < .05. During storage, total viable count was elevated significantly. The rise slope was steeper for the control relative to the other samples, which increased from 4.06 log CFU/g on the first day up to 10.4 log CFU/g on the 9th day. Additionally, as the essential oil concentration was raised, this response decreased, so that the total viable count of the sample coated with the BSM‐based coating containing the MPEO was always lower than that of the other samples on the same day of storage.

Considering the presented standards, the maximum acceptable and permitted microbial load of fresh meat is equal to 7 log CFU/g (Barzegar et al., 2020). Therefore, the microbial load of the control was under this limit only on the first day of storage. Moreover, the microbial loads of the coated sample without the essential oil and the coated one with 0.5% of the essential oil, respectively, exceeded the permitted limit after the 3rd and 6th days.

Most types of meat are highly perishable because of their high moisture and nutrient contents, as well as their nearly neutral pH. Examination of the microbial quality of meat samples ensures consumers’ health in addition to preventing economic damages (Alizadeh Behbahani and Imani Fooladi, 2018b). Statistical analysis indicated that there were significant differences between the coated samples and the control on the 3rd, 6th, and 9th days of storage. This may be caused by the difference in the pH of the samples so that the coated samples with lower pH values always had lower microbial loads relative to the control. Furthermore, numerous researchers have ascribed the lower microbial loads of the coated samples to the phenolic and flavonoid constituents, and the antioxidant activity of the essential oil (Alizadeh Behbahani, Tabatabaei Yazdi, Shahidi, Hesarinejad, et al., 2017; Seydim et al., 2006). Xiong et al. (2020) introduced carvacrol and thymol as the antimicrobial agents of MPEO. In addition, the coating acts as a barrier inhibiting the expulsion of carbon dioxide, which entails an increase in the gas content and a decrease in pH and microbial load. Incorporation of an essential oil into a coating intensifies its resistance to the expulsion of carbon dioxide (Ragab Abdallah et al., 2018). Zhang et al. (2018) and Alizadeh Behbahani et al. (2017c) reported similar results.

#### 3.6.2 Psychrotrophic count

The observed changes in the psychrotrophic count of the veal samples during refrigerated storage are depicted in Figure 5b. Mean comparison showed that there were no significant (*p* > .05) differences between the samples on the first day of storage. The shelf life of the samples was positively correlated with their psychrotrophic count. The increase slope was gentler for the coated sample containing 2% of the essential oil, compared with the other samples, and rose from 2.99 log CFU/g on the first day to 5.79 log CFU/g on the 9th day. At the same time, the most substantial variation was associated with the control. Based on the presented standards, the microbial loads of the control and the coated sample without the essential oil exceeded the permitted limit on the 6th and 9th days of storage.

The results revealed the positive effect of the coating even on the first day, and there were significant differences at 95% confidence level between the control and the coated samples during the entire storage. Pseudomonas species are among the most important psychrotrophic bacteria, which are highly aerobic and cannot grow and survive in the absence of oxygen (Alghooneh et al., 2015; Heydari et al., 2020). The BSM‐based coating acted as a barrier to oxygen diffusion and restrained the contact between the sample surfaces and oxygen (Özvural et al., 2016). Consequently, the increase rate of the growth of psychrotrophic bacteria decreased in the coated samples relative to the control. These findings conform to those of Lashkari et al. (2020) and Alizadeh Behbahani, Noshad, et al. (2020).

#### 3.6.3 Fungi count

The variations in the fungi count of the veal samples during storage are shown in Figure 5c. ANOVA demonstrated that there were no significant differences (*p* > .05) between the samples on the first day. The fungi count of all the samples was significantly elevated during storage, with the control having the most dramatic change from 0.96 log CFU/g on the first day to 3.89 log CFU/g on the 9th day. The rise in the essential oil concentration prevented drastic changes in fungi count, so that the fungi count of the coated sample containing 2% of the essential oil was lower than that of the other samples during the whole storage. Similar to Pseudomonas, fungi are also extremely aerobic and grow on the sample surface. As a result, the coating played the major role in controlling the rise in fungi count by inhibiting oxygen penetration (Alizadeh Behbahani et al., 2017c). According to statistical analyses, the control and the coated samples significantly (*p* < .05) differed from each other on the 3rd, 6th, and 9th days of storage, confirming the protective role of the coating.

#### 3.6.4 *Escherichia coli* count

The changes in the *E. coli* count of the samples during storage are summarized in Figure 5d. There were no significant differences (*p* > .05) between the samples on the first day. However, this response increased significantly during storage. The greatest change was associated with the control. The increase in the MPEO concentration restrained the rise in *E. coli* count. On the 9th day of storage, the coated samples significantly (*p* < .05) differed from the control in terms of *E. coli* count. The results also indicated that the microbial load of *S. aureus* was lower than that of *E. coli*. The difference in the cell‐wall structure of these bacteria is probably the reason behind this observation (Alizadeh Behbahani et al., 2021; Kiarsi et al., 2020; Noshad et al., 2021).

#### 3.6.5 *Staphylococcus aureus* count

The results of *S. aureus* count of the veal samples during storage at 4°C are illustrated in Figure 5e. The number of the bacteria rose significantly as time went by. At the same time, the increase rate was much lower in the BSM‐coated samples, especially those containing the MPEO, compared with the control. The control and the BSM‐coated sample containing 2% of the essential oil had the biggest and smallest changes in *S. aureus* count, respectively. The results revealed that the rate of *S. aureus* increase was significantly higher in the control than in the other samples. *S. aureus* is Gram‐positive and facultative anaerobic, that is, it does not need oxygen; but grows more properly in the presence of oxygen. Therefore, the bacterium better grew on the control, because it was not coated and was in direct contact with oxygen. Moreover, the role of the phenolic and flavonoid compounds, and the antioxidant effect of the essential oil have been pointed out in the literature (Alizadeh Behbahani and Imani Fooladi, 2018b; Barzegar et al., 2020).

### 3.7 Sensory evaluation

**FIGURE 6:**
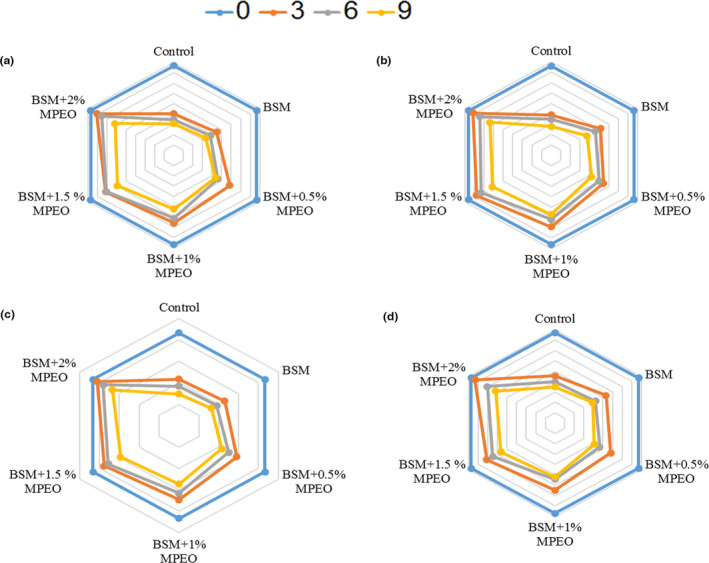
Changes in color (a), odor (b), texture (c), and overall acceptability (d) of veal samples during 9 days of storage at 4°C

The results of the sensory evaluation (color) of the veal samples during storage are shown in Figure 6a. The samples with sensory scores of more than 4 were considered acceptable. There were no significant differences between the samples on the first day. During storage, the sensory scores of all the samples were reduced, which was less pronounced in the coated samples, particularly in the coated one containing 2% of the essential oil. The sensory attribute (color) of the control and the BSM‐coated sample was not acceptable on the 6th and 9th days. On the other hand, all the coated samples containing the essential oil obtained scores of more than 4 until the 9th day.

The control had the lowest color acceptability from the panelists’ viewpoints. This may be due to the more darkness of this sample relative to the others. In the coated samples, the coating prevented the direct contact between oxygen and the sample surfaces, while there was a direct contact between oxygen and the control surface. Hence, pink oxymyoglobin was oxidized and converted into brown metmyoglobin. Additionally, the activity of aerobic yeasts on the meat surface could have brought about the emergence of brown pigments and the meat texture darkness (Suput et al., 2019; Vital et al., 2021).

The results of the sensory evaluation (odor) of the meat samples during storage are demonstrated in Figure 6b. There were no significant differences (*p* > .05) between the samples on the first day. Although the sensory scores of all the samples decreased, the control experienced the largest reduction. The odor score of the control was more than 4 merely on the first day and was not acceptable thereafter. Likewise, the score of the BSM‐coated sample without the essential oil was less than 4 on the 6th and 9th days.

It is possible that the decrease in the odor scores of the samples was caused by the products of hydroperoxides degradation. Although hydroperoxides are themselves tasteless and odorless but are basically unstable and decompose rapidly. The products of the breakdown of hydroperoxides change into aldehydes and ketones with undesirable odor and taste. The rise in the undesirable odor of the samples during storage may also be owing to the activity of microorganisms like Pseudomonas and Achromobacter (Majdinasab et al., 2020; Rezaeifar et al., 2020).

The results of the sensory evaluation (texture) of the samples during storage at 4°C are depicted in Figure 6c. There were no significant differences (*p* > .05) between the samples on the first day. Nevertheless, the differences were raised during storage. The reduction slope was steeper in the control rather than the other samples. Sensory analysis showed that all the coated samples containing the essential oil were acceptable until the 9th day. However, the control and the BSM‐coated sample free of the essential oil were not acceptable on the 6th and 9th days.

The decrease in the sensory texture score might be caused by microbial activities. Under aerobic conditions, Pseudomonas and Alcaligenes species produce slime on the meat surface, which has a negative impact on its texture. Furthermore, the growth of molds, in particular their mycelium, makes the meat texture adhesive and fluffy, adversely affecting the sensory texture score (Noshad et al., 2021). The results of the microbial tests also confirmed this fact.

The results of the sensory evaluation (overall acceptability) of the veal samples during refrigerated storage are illustrated in Figure 6d. Evaluations revealed that there was an inverse correlation between overall acceptability and storage time. At the same, the decrease in this attribute was less pronounced in the coated samples relative to the control. In spite of the coated samples containing the essential oil, which were considered acceptable until the 9th day, the control and the coated ones without the essential oil were not acceptable after the 3rd and 6th days, respectively. In conclusion, it could be declared that MPEO and the BSM‐based coating did not produce adverse effects on the sensory properties of the veal samples.

### 3.8 Correlation among physicochemical, microbiological, and sensory properties of veal samples

The correlation coefficients of the physicochemical, microbiological, and sensory properties of the veal samples are presented in Table 3. This coefficient represents the linear relationship between two variables. If the line between the variables passes through all the real points, the correlation is perfect, and if not, the correlation is imperfect. Correlation coefficient varies from −1 to 1. The coefficients of −1 and 1 represent the perfect linear correlation, whereas the values between them denote the imperfect linear correlation. The correlation coefficients of all the variables were more than 0.74. In addition, the smallest coefficient (0.74) belonged to the correlation between moisture content and ΔE. In contrast, the largest coefficient (1.00) pertained to the correlation between color and sensory texture.

**TABLE 3:** Correlation matrix (Pearson) among Physicochemical, microbiological and sensory attributes of veal

	pH	PV	TBA	Moisture	Hardness	ΔE	TVC	PTC	Fungi	*E. coli*	*S. aureus*	Color	Odor	Texture	Overall acceptability
pH	1	0.98	0.97	−0.87	−0.97	0.95	0.97	0.96	0.99	0.93	0.97	−0.90	−0.91	−0.91	−0.92
PV	0.98	1	0.98	−0.89	−0.97	0.94	0.97	0.96	0.98	0.94	0.98	−0.90	−0.92	−0.91	−0.92
TBA	0.97	0.98	1	−0.87	−0.97	0.94	0.95	0.95	0.97	0.94	0.99	−0.89	−0.89	−0.90	−0.90
Moisture	−0.87	−0.89	−0.87	1	0.90	−0.74	−0.92	−0.87	−0.89	−0.76	−0.86	0.85	0.89	0.87	0.81
Hardness	−0.97	−0.97	−0.97	0.90	1	−0.94	−0.97	−0.97	−0.98	−0.93	−0.96	0.92	0.92	0.93	0.93
ΔE	0.95	0.94	0.94	−0.74	−0.94	1	0.92	0.95	0.95	0.97	0.95	−0.85	−0.84	−0.85	−0.91
TVC	0.97	0.97	0.95	−0.92	−0.97	0.92	1	0.98	0.98	0.94	0.95	−0.91	−0.93	−0.92	−0.93
PTC	0.96	0.96	0.95	−0.87	−0.97	0.95	0.98	1	0.97	0.96	0.96	−0.92	−0.92	−0.92	−0.94
Fungi	0.99	0.98	0.97	−0.89	−0.98	0.95	0.98	0.97	1	0.94	0.97	−0.89	−0.90	−0.89	−0.91
*E. coli*	0.93	0.94	0.94	−0.76	−0.93	0.97	0.94	0.96	0.94	1	0.96	−0.83	−0.82	−0.82	−0.88
*S. aureus*	0.97	0.98	0.99	−0.86	−0.96	0.95	0.95	0.96	0.97	0.96	1	−0.88	−0.88	−0.89	−0.90
Color	−0.90	−0.90	−0.89	0.85	0.92	−0.85	−0.91	−0.92	−0.89	−0.83	−0.88	1	0.99	1	0.98
Odor	−0.91	−0.92	−0.89	0.89	0.92	−0.84	−0.93	−0.92	−0.90	−0.82	−0.88	0.99	1	0.99	0.97
Texture	−0.91	−0.91	−0.90	0.87	0.93	−0.85	−0.92	−0.92	−0.89	−0.82	−0.89	1	0.99	1	0.98
Overall acceptability	−0.92	−0.92	−0.90	0.81	0.93	−0.91	−0.93	−0.94	−0.91	−0.88	−0.90	0.98	0.97	0.98	1

## 4 CONCLUSION

Meat and meat products are always prone to microbial spoilage and oxidation, due to being composed of abundant nutrients and unsaturated fatty acids. The results of the present study demonstrated that the BSM‐based coating containing MPEO could appropriately restrain lipid oxidation, moisture loss, hardness reduction, and drastic color changes in the samples, relative to the control. According to microbial analyses, the shelf‐lives of the coated sample free of the essential oil and the coated ones containing the essential oil at 0.5%, 1%, 1.5%, and 2% were, respectively, extended up to 3, 6, 9, 9, and 9 days, compared with the control. Moreover, the coating containing the essential oil had no unfavorable effect on the sensory attributes of the veal samples. In conclusion, the BSM‐based coating containing MPEO could be utilized to package and extend the shelf life of meat products as well as other fresh food products.

## CONFLICT OF INTEREST

The authors declare no conflict of interest.

## AUTHOR CONTRIBUTION

**Hadi Tanavar:** Conceptualization (equal); Data curation (equal); Formal analysis (equal); Investigation (equal); Methodology (equal); Software (equal); Writing‐original draft (equal). **Hasan Barzegar:** Funding acquisition (equal); Project administration (equal); Supervision (equal); Writing‐review & editing (equal). **Behrooz Alizadeh Behbahani:** Data curation (equal); Formal analysis (equal); Validation (equal); Writing‐original draft (equal). **Mohammad Amin Mehrnia:** Formal analysis (equal); Software (equal); Writing‐original draft (equal).

## ETHICAL APPROVAL

The authors declare that this study did not involve human or animal subjects, and human and animal testing were unnecessary in this study.

## Data Availability

The data that support the findings of this study are available from the corresponding author upon reasonable request.
